# Persistent use of nitrous oxide for anaesthesia in European hospitals despite its harmfulness to the climate – how emission taxation can achieve the coupling of cost-effectiveness and climate protection: observational study

**DOI:** 10.1186/s12913-023-10307-y

**Published:** 2023-12-11

**Authors:** Ferdinand Lehmann, Christian M Schulz, Dominik Leicht, Scott Brady, Anna Fuhrmann, Jens Prütting, Max Georg Hügel, Daniel Hörr, Michael Sander

**Affiliations:** 1 Klinik für Anästhesiologie, operative Intensivmedizin und Schmerztherapie, UKGM Standort Gießen, Justus-Liebig-Universität, Gießen, Germany; 2 Deutsche Allianz für Klima und Gesundheit e.V, Berlin, Germany; 3 Health Care Without Harm (HCWH) Europe, Brussels, Belgium; 4 Bucerius Law School gGmbH, Hochschule für Rechtswissenschaft, Hamburg, Germany; 5 Geschäftsbereich Technik und Bau, UKGM, Standort Gießen, Germany

**Keywords:** Sustainability, Carbon footprint, General anaesthesia, Nitrous oxide, Emission taxation

## Abstract

**Background:**

Health care has the intrinsic obligation to preserve health. This concept is also applicable to planetary health. Nitrous oxide (N_2_O) lacks clinical indications in modern anaesthesia, while it is a high-potential greenhouse gas. Its seemingly low cost contrasts with the consequential externalised socio-economic costs due to its contribution to the climate crisis, which is approximately €698 per emitted ton of CO_2_ equivalent. This difference can be internalised through emission taxation. In this study, we aim to evaluate how much N_2_O – total amount and converted to CO_2_ equivalent – is used at a German university hospital and compare this amount to that used at European hospitals. Furthermore, how the cost of N_2_O usage changes under different emission taxation scenarios is calculated.

**Methods:**

This trial was a retrospective observational study at a German university hospital with approximately 1,250 beds between 2016 and 2020. Additionally, five European hospitals from the Health Care Without Harm Network were used for comparison from a European perspective. The main outcome parameters were the amount of N_2_O used, in total and converted to CO_2_ equivalent, and the total cost at emission taxation of €0, €25, €55 and €698 per ton CO_2_ equivalent.

**Results:**

At the peak, 2,104 tCO_2_ equivalent in N_2_O was emitted in 2019. The actual cost was €14,040 in this year, while the corresponding socio-economic damage due to the climate crisis was almost €1.5 million. Other European hospitals showed comparable amounts of emissions.

**Conclusions:**

The annual peak amount of emitted N_2_O corresponded to the total annual greenhouse gas emission of 188 people in Germany. To achieve a drastic reduction in use, the abandonment of recommendations by anaesthesiologic societies appears necessary, in addition to an internalisation of future costs via emission taxation, which will cause inadequate cost for a medication without relevant benefit or indication. To that end, the inclusion of health sector emissions within national or international greenhouse gas taxation, for example, the European Union Emissions Trading System, appears necessary and expedient in view of the urgent need to address the ecological transformation.

**Trial registration:**

The trial was registered with the German Clinical Trials Register, identifier DRKS00024973 on 12/04/2021.

## Background

 The climate crisis is the greatest threat to human health in the 21st century. Worldwide, vital social and environmental aspects of health like clean air, safe drinking water, food security and shelter are at risk and affect especially countries with weak health care infrastructure [1]. The direct and indirect consequences of the climate crisis are already leading to an increase in the burden of disease worldwide, including in Europe [2]. The health care systems in Western countries contribute 5% of greenhouse gas emissions, thus fuelling the climate crisis [3]. For the health sector to be able to provide health care with minimal side effects (primum nihil nocere), health care facilities such as hospitals themselves have a responsibility to rapidly reduce emissions [4].

Minimising the use of the inhalational anaesthetic nitrous oxide (N_2_O) can make a relatively simple contribution with relevant impact. As a potent greenhouse gas (CO_2_ equivalent [CO_2_e] 298 over 100 years) emitted especially by agriculture, nitrous oxide is responsible for approximately 6.4% of the global greenhouse effect [5]. Within the Western health sector, its share in total emissions is approximately 2%, compared with other volatile anaesthetics that add another 0.5% [3, 6, 7]. Notably, sufficient data is mainly delivered by the British health care sector, while detailed data from other European health care systems or hospitals are scarce. Although alternatives are available for the vast majority of clinical applications and in spite of relevant side effects and contraindications, nitrous oxide is still frequently used in anaesthesia, not least because of its seemingly low cost [8, 9]. In view of the lack of cogent indications, the significant quantity of emissions and the immense follow-up costs due to climate crisis, the uncritical use of nitrous oxide should be questioned, as should its provision, which is presumably often associated with leakage [10].

Thus far, within the European Union (EU), the consequential socio-economic costs of greenhouse gas emissions are reflected only within the transport, real estate, production and energy sectors through CO_2_ emission taxation [11]. Since the direct emissions caused by the health sector have thus far been excluded from the emission taxation, the price of inhalational anaesthetics currently remains low despite the future damage to health, the environment and the economy due to the climate crisis [12].

The aim of this study is to illustrate the amount of emitted nitrous oxide, its current costs and how the economic viability of the drug could change if the follow-up costs were internalised through CO_2_ pricing, using a German university hospital as an example. For the purpose of comparability within the European context, data on the consumption of nitrous oxide by other European hospitals from the Health Care Without Harm (HCWH) clinic network are used. This network offers European health care providers the opportunity to evaluate the individual greenhouse gas emissions and other environmentally relevant indicators with professional support.

## Methods

The study design was a retrospective observational trial. Ethical approval for this study was provided by the Ethics Committee of Justus-Liebig University, Gießen, Germany (Chairperson Prof Dr H Tillmanns) on 22 February 2021, identifier AZ 17/21, and the study adhered to the latest version of the Declaration of Helsinki. The trial was registered on 12/04/2021 with the German Clinical Trials Register, identifier DRKS00024973. The data on used N_2_O were collected for the Department of Anaesthesiology, Surgical Intensive Care and Pain Therapy, University Hospital Gießen (UKG), Gießen, Germany, a maximum-care hospital with approximately 1250 beds. Depending on the workplace, nitrous oxide was supplied to the anaesthesia machine by either central supply (via containers with 450 kg) or locally installed pressure bottles (7.5 or 37.5 kg filling). The hospital’s medical technology department, which is responsible for maintaining the system, provided data on maintenance costs, the quantity of usage and the price of various forms of nitrous oxide. To avoid the inclusion of data on N_2_O used by the obstetrics department, records of nitrous oxide orders by both departments were cross-checked for potential overlap. Furthermore, it was ensured that the delivery of N_2_O in the obstetrics department remained different over the study period. Additionally, it was ensured that no other department used N_2_O. The data from 2016 to 2020, which is the available time frame of the actual hospitals’ digital ordering systems, were mapped. The number of anaesthetic procedures involving nitrous oxide was determined using the department’s internal documentation software (IMESO-IT, Gießen, Germany). Further specific data on usage concerning the administering anaesthesiologist, surgical setting and indication were not collected to prevent individual identification. In addition, the costs of the direct emission of nitrous oxide in CO_2_ equivalent (CO_2_e) were calculated using the current taxation price in Germany as of 2021 (€25/tCO_2_), the target price for 2025 (€55/tCO_2_) and the calculated anticipated socio-economic costs (calculated by the German Federal Environment Agency to be €698/tCO_2_) [13, 14]. European emission taxation is based on certificate trade and is therefore subject to relevant fluctuations. As it varies within comparable margins of the German fixed taxation system, fixed values were used for calculation. In addition, HCWH presented data from five European hospitals for 2019 and 2020. These represent all hospitals in the HCWH network that continuously use N_2_O and provided data on usage. In order to compare the emissions of different hospitals, emissions per hospital bed were calculated. Additionally, the collaboration with other experts within the network of ecological transformation of the health care system (Bucerius Law School Hamburg and Deutsche Allianz Klimawandel und Gesundheit e.V.) was established to add further perspectives on the subject. The evaluation was carried out purely descriptively using Microsoft Excel (Microsoft, Redmond, USA).

## Results

The N_2_O consumption at UKG specified by the type of supply, the total annual consumption and the corresponding CO_2_ equivalent were calculated (Table 1). Data show usage of nitrous oxide over the entire period. After an incline from 2016 and a peak in 2019 the usage declined in 2020 because of lower use via piping system. At maximum, 2,104 tCO_2_ equivalent in N_2_O was emitted in 2019. Within the study period, anaesthetic procedures using nitrous oxide decreased from 8.1 to 3.6%.

**Table 1:** Quantity of nitrous oxide used at university hospital Gießen

Year	Number of pressure bottles 7.5 kg	Number of pressure bottles 37.5 kg	Number of pressure bottle containers 450 kg	Total consumption in kg	Total tCO_2_e emissions due to nitrous oxide
2016	12	3	5	2,460	731
2017	20	18	7	4,035	1,185
2018	10	8	9	4,440	1,319
2019	11	6	15	7,155	2,104
2020	11	6	10	5,055	1,434

The total annual costs of nitrous oxide at UKG, excluding maintenance, depending on CO_2_e pricing were mapped between 2016 and 2020 (Table 2). In addition to the costs of nitrous oxide, there were approximately €600 per year in maintenance costs for the central system, which were calculated proportionately to the maintenance contract by the medical technology department. The actual cost for N_2_O was €14,040 in 2019, while the corresponding theoretic socio-economic damage due to the climate crisis was almost €1.5 million. Even if only the German emission taxation with 55 € per tonne CO_2_e was applied, an annual price in 2019 of almost 130,000 € appears clearly relevant for a medication that is not needed.

**Table 2:** Annual costs of nitrous oxide depending on the pricing regimen applied, based on emissions university hospital Gießen

Year	Total tCO_2_e emissions due to nitrous oxide	Total cost if 1 tonne CO_2e_ = €0	Total cost if 1 tonne CO_2e_ = €25 (price in Germany in 2021)	Total cost if 1 tonne CO_2e_ = €55 (price in Germany in 2025)	Total cost if 1 tonne CO_2e_ = €698 (calculated socio-economic damage)
2016	731	4,907	23,179	45,104	515,037
2017	1,185	7,944	37,558	73,094	834,760
2018	1,319	8,810	41,776	81,336	929,228
2019	2,104	14,040	66,618	129,712	1,482,028
2020	1,434	9,575	45,390	88,369	1,009,554

Total price without annual maintenance costs of approximately €600. Net prices. CO_2_e – CO_2_ equivalent

In total, five datasets of nitrous oxide consumption in 2019 and 2020 from the HCWH network were available, including five different European hospitals on the Iberian Peninsula and the British Isles (Table 3). The Hospital’s sizes varied between 240 and 6500 beds. Comparison was made with available data from UKG during the same periods. Nitrous oxide emissions per bed and year ranged between 0.4 and 8.0 tCO_2_e with UKG emission data sets at 1.1 and 1.7 tCO_2_e thus showing that German emission data are within margins of other European health care systems emissions.

**Table 3:** Comparison of 6 different European hospitals by total nitrous oxide use and relative use per hospital bed (excluding obstetrics, HCWH hospitals anonymised)

Hospital	Region	Year	Number of hospital beds (rounded)	Total tCO_2_e emissions due to nitrous oxide	tCO_2_e emissions per bed and year
HCWH record #1	British Isles	2019	2300	1,585	0.7
HCWH record #2	Iberian Peninsula	2019	350	139	0.4
HCWH record #3	Iberian Peninsula	2019	240	1,928	8.0
HCWH record #4	British Isles	2020	2800	3,919	1.4
HCWH record #5	Iberian Peninsula	2020	6500	2,869	0.4
UKG	Germany	2019	1250	2,104	1.7
UKG	Germany	2020	1250	1,434	1.1

*HCWH *Health Care Without Harm, *CO*_2_eCO_2_ equivalent, *UKG *University Hospital Gießen

## Discussion

In the present study, we modelled the costs of nitrous oxide use at a German academic anaesthesiology department under (I) current conditions, (II) in the context of an assumed introduction of emission taxation for medical greenhouse gases, and (III) with the inclusion of subsequent socio-economic damages in the context of the climate crisis.

The consumption of nitrous oxide in the anaesthesiology department at UKG peaked in the pre-pandemic year 2019, and the amount translated into the individual carbon footprint of approximately 188 people in Germany (at 11.2 tCO_2_e/year and person, Federal Environment Agency CO_2_ calculator as of 2020). This is approximately the number of physicians and nurses of this department, while the drug is used in less than 10% of procedures. With a CO_2_e price of €698 per tonne, which captures the socio-economic damage caused by this emission, nitrous oxide usage corresponds to annual additional costs of almost €1.5 million for University Hospital Gießen in 2019. Even with pricing at only the planned €55 per tonne of CO_2_e in 2025, annual additional costs of almost €130,000 would not justify the use of nitrous oxide either economically or medically. Based on these findings, the availability of nitrous oxide in UKG was massively restricted. An exemplary comparison with other European hospitals from the HCWH network shows that comparable quantities of nitrous oxide are used in different European health care systems. This implies that pan-European solutions for emission reduction are preferable. A more extended comparison with other hospitals and health care systems was not possible as no such data could be identified by the research team.

The fluctuations in annual usage and the differences between hospitals may be due to variable leakage of the pipe system, changes in staff or consequences of changes in recommendations and guidelines, which fall beyond the scope of this article. Furthermore, discussions should be aimed at general mechanisms that help to drastically reduce the medical emission of N_2_O equally in different hospitals and health care systems. Reasons for the persistent use can be limited availability of anaesthesia coverage as it can be safely administered by nursing staff. Also, in dentistry and obstetrics the use of N_2_O is common, but with adequate alternatives readily available [8, 15, 16]. Additionally, the use might persist due to traditional and historical reasons in certain hospitals with underestimation of its climate potential. It may also be considered to continuously use N_2_O in countries with reduced financial means as a cheap hypnotic, especially as the World Health Organization considers it essential. Here it should be emphasised that usage without an adequate gas suction system (which is expensive in operation) N_2_O bears relevant side effects especially for the medical staff which limits its use.

Of course, the simultaneous use of nitrous oxide reduces the consumption of volatile anaesthetics, which is a frequently used argument against the abandonment of nitrous oxide. In view of the greenhouse gas potential of N_2_O (CO_2_e 298) with 50% in the fresh gas flow, compared to sevoflurane (CO_2_e 130) at approximately 2–3% in fresh gas, the usage of nitrous oxide will cause higher total emissions rather than saving enough sevoflurane to lower them. Here, desflurane, with a greenhouse gas potential of over 2500, will not be further considered, as it is obsolete for use as a modern anaesthesia in times of the incipient climate crisis. Additionally, it lacks patient-relevant advantages compared to sevoflurane. Its usability appears very limited without widely available complete adsorption by activated carbon filters and subsequent treatment [17].

### What are the options for reducing emissions?

It is doubtful that a complete political ban on nitrous oxide would be accepted by European anaesthesiologists [18, 19]. To achieve the necessary drastic reduction in emissions caused by nitrous oxide use in a timely manner, various approaches appear feasible.

1) Anaesthesiologic societies are responsible for translating the climate relevance of anaesthetic agents into guidelines, recommendations and disclaimers. It must be emphasised that as a health system, we are currently not fulfilling our intrinsic task of primarily avoiding damage to people’s health in the context of the climate crisis. Additionally, a comprehensive introduction of sustainability in training and teaching environments would be useful.

Currently, many international anaesthesiologic societies, such as the American Society of Anesthesiologist (ASA) and European Society of Anaesthesiology and Intensive Care (ESAIC), have not yet come out clearly against the use of nitrous oxide in anaesthesia. Rather, they have only referred to its climate relevance as a sidenote [9, 20, 21]. The British National Health Service (NHS) addresses the problem and focuses mainly on education and on reducing system losses, while the Société Française d’Anesthésie et de Réanimation (SFAR) in France and the Berufsverband Deutscher Anästhesisten (BDA) and Deutsche Gesellschaft für Anästhesiologie und Intensivmedizin (DGAI) in Germany explicitly advise against the use of nitrous oxide [10, 22, 23]. The World Federation of Societies of Anaesthesiologists opposes a complete abandonment but recommends limiting its use to non-further specified cases [19].

Despite the current resistance in banning N_2_O from anaesthesiologic practise, stating a contraindication within guidelines can be considered an effective tool. In the authors’ view it is appropriate considering the consequential damage and the lack of patient-relevant treatment benefits. The legal consequences of establishing a contraindication of the use of nitrous oxide in general anaesthesia, are of interest (Table 4). Although the preparation of guidelines and its legal implications is complex, discussion within anaesthesiologic societies should be extended here.

**Table 4:** Legal consequences of a contraindication of nitrous oxide

From the perspective of legal economics, it should be noted that anchoring a medical contraindication of nitrous oxide in the guidelines of anaesthesiologic professional societies can lead to health care providers refraining from its use to avoid liability risks.[1] The reason is that professional guidelines indirectly influence the assessment of the medical standard according to which treatment must be carried out to avoid claims for damages (in Germany: section 630a (2) of the German Civil Code). In addition, effects on financing law in the area of statutory and private health insurance are to be expected in the medium term, as the determination of standards in these legal fields follows comparable criteria with regard to the necessity of a medical intervention.[2] As an effect, a significant emission reduction through nitrous oxide would be probable. [1] Cf. Prütting, J. (2020). Rechtsgebietsübergreifende Normenkollisionen (Conflicts Of Norms Across Legal Fields), Mohr Siebeck, pp. 256, 259 [2] Section 2 (1) sentence 3 and section 12 (1) of Book V of the German Social Code as well as section 192 (1) of the German Insurance Contract Act; cf. Peters (2021), Kasseler Kommentar zum Sozialgesetzbuch V (Kassel Commentary on Book V of the Social Code), C.H.Beck, section 2, margin note 4; Kalis (2017), Münchener Kommentar zum Versicherungsvertragsgesetz (Munich Commentary on the Insurance Contract Act). 2nd edition. C.H.Beck, section 192, margin note 23 et seq.	

2) Technical solutions for the substantial N_2_O losses that occur via the supplying gas systems are necessary, as are approaches such as the catalytic destruction of nitrous oxide after its use [10]. However, these solutions and approaches are cost-intensive or not market-ready solutions and can only constitute supportive measures where the abandonment of nitrous oxide usage is not possible.

3) One comprehensive approach to reducing unnecessary greenhouse gas emissions from the health system is the “internalisation of climate impact costs” demonstrated in this article. The inclusion of direct medical greenhouse gas emissions in the European Union Emission Trading System or that of individual countries would be one such possibility. Such an inclusion would be feasible without creating new legal framework structures. A legal perspective on the demand for emission taxation is also to be considered (Table 5), which supports this idea and emphasizes its timely advantages.

**Table 5:** Legal background to an emission tax on nitrous oxide in the health sector

The European Union Emissions Trading System has been covering nitrous oxide as a greenhouse gas since 2013 but only in the energy and industry sectors. Individual member states, such as Germany, limit their CO_2_ tax to the areas of heat and transport; thus, they do not include the health sector [1]. Therefore, consideration should be given to extending the tax obligation to nitrous oxide in the health sector at the European level or at the national level by adapting the German Fuel Emissions Trading Act [2] and comparable regulations in other countries. Insofar as gaps remain in European emissions trading regulations, national emissions trading can also take effect (section 1 of the German Fuel Emissions Trading Act). German law refers to the list of goods in the European Combined Nomenclature [3]; thus, it would only have to be expanded to include the nitrogen oxides already listed in the nomenclature (CN 2811 29 30). From the authors' perspective, the fiscal way to curb the use of nitrous oxide in the health system seems more efficient than a regulation based on national health law. In Germany, in particular, this would otherwise require lengthy procedures and the involvement of a wide range of interest groups and stakeholders in the health care system, for example, in the case of an adjustment of the hospital treatment guideline of the Joint Federal Committee to exclude nitrous oxide-based methods [4] or an adjustment of billing and remuneration regulations. Regardless, a single pan-European solution would not be achievable via health law due to the different legal structures of the EU member states. [1] German Emissions Trading Authority (2019), Factsheet European Emissions Trading 2013-2020. Retrieved from https://www.dehst.de/SharedDocs/downloads/DE/publikationen/Factsheet_EH-2013-2020.pdf?__blob=publicationFile&v=7 [2] Section 2 (1) and Annex 1 of the Act on National Certificate Trading for Fuel Emissions (Fuel Emissions Trading Act) of 12 December 2019, last amended on 3 November 2020. [3] Combined nomenclature as referred to in Article 1 of Council Regulation (EEC) No 2658/87 of 23 July 1987 on the tariff and statistical nomenclature and on the Common Customs Tariff, as amended by the corrigendum to Commission Implementing Regulation (EU) 2021/832 of 12 October 2021, OJ EU L 414, 19 November 2021. [4] Directive of the German Federal Joint Committee on examination and treatment methods in hospitals in the version of 21 March 2006, last amended on 16 September 2021, BAnz AT 25. November 2021 B2.	

The effectiveness of such an approach becomes evident, when considering the mechanisms of selection of medical products. Currently, the selection of medical products in Germany is mostly based on their cost-effectiveness, i.e., the relationship between costs and medical benefits, but not their climate footprint [24]. A coupling of cost-effectiveness and sustainability via emission taxation on medical products like N_2_O appears to be a comprehensive way to achieve an internalisation of climate impact costs – therapies that cause significant emissions will become pricier based on their harmfulness to the climate and environment. By (at least partially) reflecting the anticipated socio-economic costs due to the climate crisis caused by the emission of greenhouse gases such as nitrous oxide, the changed cost-effectiveness of certain medical therapies would lead to a significant adjustment in usage. From the perspective of the market economy, a significant increase in cost would not be acceptable for a drug such as nitrous oxide, which does not offer relevant advantages in treatment. Its use would become obsolete not only medically but also economically. It should be emphasised that a pan-European solution to reduce emissions from health systems should be sought. As the emerging climate crisis demands for timely and effective measures to reduce greenhouse gas emissions, lengthy legal adjustments should be avoided.

From 2024, according to the European Union’s Corporate Sustainability Reporting Directive (CSRD), the vast majority of European hospitals will be obliged to publish an annual sustainability report [25]. The expected transparent quantification of direct emissions such as nitrous oxide, which is crucial but currently difficult as presented, may show how extensive health care’s contribution to the climate crisis is in detail. However, the health system should address the question of which emissions can be avoided and which paths can be taken to make health preservation, climate protection and economy compatible at an earlier stage. Following the recommendations of international medical organisations, crucial and impactful steps include measures of energy saving, usage of renewable energy and preference of low-emissive medical products [6, 7, 19, 23]. However, some impactful interventions are extensive and cost-intensive. Timely ecological transformation of international health care systems may reduce the already present threat for human health by the climate crisis and lower the extent of necessary adaption measures [2].

### Next steps and future directions

Undoubtedly, climate crisis obliges all sectors to a significant reduction of their greenhouse gas emissions. In the medical sector, besides direct emissions via medical gases or fossil fuels for vehicles, heating and electricity, large proportions of emissions lie within the life cycle of medical products. Between 50 and 75% of health care’s total emissions are caused by raw material extraction, manufacture, transport, usage and disposal of medication, disposables/consumables and equipment [26]. Quantification of a product’s ecological harm via standardised life cycle assessment (LCA) constitutes the necessary basis to identify ecologically preferable products. For example, the use of renewable energy and biodegradable plastics as well as a reduction of multilayer packaging and mixed-material-products can reduce emissions [19]. In order to reach a net zero emission health care system, policy makers have to find ways to favour ecologically preferable products and medical practises. Such a way may be via emission taxation as proposed in the article as well as the obligation of preference of the most ecological product. However, this choice needs to be based on robust data of the potential ecological hazard posed by products thus requires extensive and independent research to generate high quality LCAs.

In order to reduce health care’s carbon emission from heating, electricity and transportation, the use of renewable energy needs to not only be the most environmentally sustainable option but also the most economic one. Again, the coupling of cost-effectiveness and climate protection appears to be preferable as current practise of the EU CO_2_ emission taxation shows. The planned increase of emission prices is essential to decrease the use of fossil fuels [11].

In addition, principles of environmental sustainability have to be prioritised within research, professional recognition and quality improvement programmes [19]. Also, all interventions to reduce emissions have to consider different economic, political and social systems in order to achieve a global solution for climate and other ecological crises [27, 28]. Only by international cooperation and timely interventions by policy makers and researchers global warming can be limited to an extent of 1.5–2 degree. In case of failure to do so, extensive measures of adaptation with increasing morbidity and mortality as well as spread of so far “uncommon” infectious diseases have to be implemented [2, 29].

### Limitations and potential bias

Firstly, this work is limited by the simplified price assumption of CO_2_ equivalent. In the EU, certificate trading is implemented, while in Germany, the fixed target price mentioned above is used. Further, the United Kingdom has implemented its own CO_2_ pricing system (UK ETS). Therefore, the costs are to be understood only as examples and are potentially biased, and they reduce the generalisability of the presented data. Additionally, the calculation of socio-economic costs caused by greenhouse gas emissions by the German Federal Environment Agency varies as the climate crisis progresses. The calculation should be understood as an illustration of the magnitude of the damage rather than the precise value, which is another source of a potential reduction in the generalisability of the results to other scenarios and future times.

Secondly, sufficient data on the extent of usage of nitrous oxide within Europe are incomplete for different reasons. The NHS can provide the most detailed data due to its structure while other European health care systems do not, presumably because of the lack of central surveys. Also, the usage of N_2_O facing its side effects and greenhouse gas potential does not necessarily reflect modern anaesthetic practise thus might be influencing surveys. This led us to the approach to get information via the HCWH network, which again is biased because the participating hospitals do have interest in the area of ecological sustainability. Still, it succeeds to show that the use of N_2_O is present even within these transformative networks, suggesting that it is even more present outside of it. This implies that collective European interventions are of use. Another limitation is the lack of further characteristics and detailed information about the compared hospitals which were kept anonymous by HCWH regulations.

Furthermore, it should be noted that the share of medical nitrous oxide emissions is relatively small compared to those caused by agriculture. However, medical N_2_O emissions are almost completely avoidable and represent an essential, timely and simple step on the way to a climate-neutral health system.

Lastly, facing the high secondary costs due to the carbon potential of nitrous oxide, a conceivable theoretical potential for optimising anaesthesia management with N_2_O was not considered. An analogy to xenon, which, as an almost optimal volatile anaesthetic, could offer advantages over common drugs, can be drawn. However, xenon is generally not used because the costs do not outweigh the benefits. This also applies to N_2_O in the case of emission pricing, which may cause bias.

## Conclusion

In summary, this work shows that even with a low use of nitrous oxide in anaesthesiology departments, it causes significant greenhouse gas emissions. This applies to hospitals throughout Europe. To avoid unnecessary emissions of medical greenhouse gases, an internalisation of the consequential socio-economic costs causes these therapies to have a low cost-effectiveness, which represents a simple and timely intervention. Internalisation can currently be achieved through the adequate pricing of emitted CO_2_ equivalent and, thus, the depiction of the upcoming cost caused by the climate crisis. To that end, the inclusion of health sector emissions in national or international greenhouse gas taxation, for example, the European Union Emissions Trading System, appears necessary and expedient in view of the urgent need to address the ecological transformation. Research on the effect of present emission taxation on the usage of anaesthetics such as nitrous oxide will be needed to prove the usefulness of this taxation.

## Data Availability

The datasets used and/or analysed during the current study are available from the corresponding author upon reasonable request.

## Abbreviations

ASA: American Society of Anesthesiologist
BDA: Berufsverband Deutscher Anästhesisten
CO_2_e: CO_2_ equivalent
CSRD: Corporate Sustainability Reporting Directive
DGAI: Deutsche Gesellschaft für Anästhesiologie und Intensivmedizin
ESAIC: European Society of Anaesthesiology and Intensive Care
EU: European Union
HCWH: Health Care Without Harm
LCA: Life Cycle Assessment
NHS: National Health Service
N_2_O: Nitrous Oxide
SFAR: Société Française d'Anesthésie et de Réanimation
UKG: University Hospital Gießen
